# Supplementary material for: Carrying out embedded implementation research in humanitarian settings: A qualitative study in Cox's Bazar, Bangladesh
Source: PLoS Med. 2020 Jul 16;17(7):e1003148. doi: 10.1371/journal.pmed.1003148 (PMC7365392; doi:10.1371/journal.pmed.1003148)
Supplement: S1 COREQ Checklist — (DOCX) [file pmed.1003148.s001.docx]

**Supporting Information**

Consolidated criteria for reporting qualitative studies (COREQ): 32-item checklist

| **No** | **Item** | **Guide questions/description** |
| --- | --- | --- |
| **Domain 1: Research team and reflexivity** | | |
| Personal Characteristics | | |
| 1. | Interviewer/facilitator | ASMS, AH, MS |
| 2. | Credentials | ASMS (PhD), ABS (PhD), DJ (PhD), PR (PhD), AH (DrPH), MS (PhD) |
| 3. | Occupation | ASMS (Health Specialist), ABS (Senior Health Specialist), DJ (Senior Advisor-Health), PR (Regional Adviser Health), AH (Immunization and Research Specialist), MS (Professor of Public Health) |
| 4. | Gender | One interviewer was female and two were male. |
| 5. | Experience and training | ASMS, AH and MS have extensive experience with conducting in-depth interviews for qualitative research studies; All have published extensively. |
| Relationship with participants | | |
| 6. | Relationship established | None. |
| 7. | Participant knowledge of the interviewer | Few participants were known to the ASMS and MS. |
| 8. | Interviewer characteristics | All interviewers are public health researchers and are particularly interested in qualitative methods |
| **Domain 2: Study design** | | |
| Theoretical framework | | |
| 9. | Methodological orientation and Theory | Qualitative methods and thematic analysis |
| Participant selection | | |
| 10. | Sampling | Purposive sampling |
| 11. | Method of approach | We approach representatives from UN organizations, NGOs, INGOs, and Government through colleagues from UNICEF Cox’s Bazar Field office. After appointment, we went to their offices to conduct the interviews. For other interviewers, we communicated with them over phone. Once they agreed to take part in the study, we approached them and face to face interviews were done by two researchers. |
| 12. | Sample size | 12 |
| 13. | Non-participation | None. We purposively selected participants for interview. And all participants agreed to give an interview after we approached them. |
| Setting | | |
| 14. | Setting of data collection | Offices |
| 15. | Presence of non-participants | No |
| 16. | Description of sample | See Table 1 |
| Data collection | | |
| 17. | Interview guide | An interview guide was drafted, piloted, and revised. |
| 18. | Repeat interviews | None |
| 19. | Audio/visual recording | Seven interviews were audio-recorder and for five interviews extensive field notes were taken |
| 20. | Field notes | Yes |
| 21. | Duration | 25-60 minutes |
| 22. | Data saturation | Yes |
| 23. | Transcripts returned | No |
| **Domain 3: Analysis and findings** | | |
| Data analysis | | |
| 24. | Number of data coders | Four |
| 25. | Description of the coding tree | A coding tree was not developed. |
| 26. | Derivation of themes | Theme were derived from the data based on thematic analysis approach |
| 27. | Software | No |
| 28. | Participant checking | Not with the participants of the first phase. ASMS and MS sought informal feedback from the second phase participants to check the validity of the results. |
| Reporting | | |
| 29. | Quotations presented | Yes |
| 30. | Data and findings consistent | Several relevant quotations used to illustrate findings. |
| 31. | Clarity of major themes | Yes |
| 32. | Clarity of minor themes | Yes |
