# Supplementary material for: Carrying out embedded implementation research in humanitarian settings: A qualitative study in Cox's Bazar, Bangladesh
Source: PLoS Med. 2020 Jul 16;17(7):e1003148. doi: 10.1371/journal.pmed.1003148 (PMC7365392; doi:10.1371/journal.pmed.1003148)
Supplement: Attachment — Submitted filename: shahabuddin.pdf [file pmed.1003148.s002.pdf]

This paper describes a study of the processes behind an implementation research project in Bangladesh.

I was asked for a methodological report and I interpret that to include all aspects of the design and conduct of the study. The version I received did not have page numbers. My numbering has the abstract starting on page 2

1 Points of detail

Page 4 It might be helpful to define upazilas. By all means keep the word in the original too. I know we can look it up in Wikipedia of course.

Page 12 '... BRAC team faced several challenges while collecting data ...' does not seem to tell us what those challenges were.

Page 14 The paragraph starting 'Methods of IR' does not seem to be a finding from the study but rather a generic description of IR. It could be omitted without loss of impact.

Page 15 When the authors call for an agile approach do they mean in the sense it is used in software development with roles like a scrum master or is it some other meaning? If it is the sense used in software development then it may need further explanation.

2 Points of more substance

2.1 What is specific to IR and humanitarian setting

The authors have worked in a challenging setting and are trying to provide background help for people carrying out implementation research in humanitarian settings. But I would question how much of what they have found is limited to those settings and how much applies more generally to any type of research in any resource–poor setting. The article is quite short and more detail might help to answer my concerns. One thing I miss is any quotes from the interviewees which I feel would help to illuminate things for the reader rather than rely on the auhtors' summaries of what people said.

I take the authors' five themes in turn.

Unique and complex context [. . .] multiple actors

This applies to implementation research generally I think but is it specific to humanitarian contexts?

Adaptation to frequent changes [. . .]

I think anyone who has tried to carry out research based in a large organisation anywhere in the world would have faced the issue of constant changes undermining the organisation and inadvertently sabotaging the research project.

Difficulty accessing the camps [. . .]

This is true but not specific to implementation research. Any epidemiological study in rural areas with rainy seasons experiences this sort of problem. For instance in a study of a consecutive series of pregnant women and their child in rural Ethiopia our interviewers complained quite vocally about having to make visits to the more remote locations during the rainy season.

Language and barriers to access to quality information

This too seems to me to be a general issue. If you want to work with monoglot speakers of language X you have to learn language X or enlist staff who do speak it. To be fair I have read authors claiming as an advantage that they hired people who spoke the local language so perhaps it is not as obvious to others as it is to me.

Need for experienced in–country research partners

Again this is quite general. Things are slowly changing but I can remember the days when I received articles to review with not a single in–country author. Even for laboratory studies this seems a drawback. The point is still worht making as change is slow but again I question how specific it is to the context which the authors are working in.

2.2 What exactly was the interview content for all the stakeholders?

I am not clear what the interviews with the NGO were intended to reveal, what their content was, and what the outcome is. Most of the findings seem to come from the work with the BRAC team. This does seem another area where some example quotes might help us to understand what happened.

3 Summary

I have no problems with the advice the authors give and I am sure it will ring true with many people who have worked in low resource settings but I wonder how specific it is to implementation research in humanitarian settings.

Michael Dewey
