# Supplementary material for: Carrying out embedded implementation research in humanitarian settings: A qualitative study in Cox's Bazar, Bangladesh
Source: PLoS Med. 2020 Jul 16;17(7):e1003148. doi: 10.1371/journal.pmed.1003148 (PMC7365392; doi:10.1371/journal.pmed.1003148)
Supplement: Attachment — Submitted filename: Response to Reviewers.docx [file pmed.1003148.s003.docx]

*** Reviewer #1:**

The authors are correct in identifying both the gap and the potential for research in a humanitarian emergency. They are also correct in identifying the unique situation in which the Rohingya in Bangladesh find themselves, sheltered in a set of camps that form the largest migrant population in the world. Although they briefly explain the fundamentals of embedded implementation research and proceed to establish the difficulties of conducting such research in a crisis situation, it would seem prudent to outline the process for conducting a successful IR project at the outset, and then later compare it to their own observations.

**Response:** Many thanks and grateful to the reviewer for all valuable comments. In terms of the process of conducting embedded implementation research (IR), different organization follow different approach. UNICEF has been implementing an approach since 2015 which we have briefly mentioned in the manuscript. Please see the revised version of the manuscript where in the ‘Introduction’ we have the following information.

“Embedded IR bridges the gap between research and policy by embedding relevant decision-makers and programme implementers (programme managers, front-line health workers etc.) in the process of research and dealing with issues related to health systems that have relevance in programmes and policies (1–6). This approach focuses on the questions identified by decision-makers and programme implementers and ensures that knowledge generated is relevant.

Although the magnitude of the current crisis is alluded to (in terms of migrant numbers), its scale can be further quantified by mentioning the total number of camps present as well as the total number of primary health-care facilities in them. This data may help the authors further explain some of the difficulties they faced in conducting this project. However, this will highlight the very small sample size they have taken in Phase 1.

**Response:** We have recently published a relevant article where description of the camp settings in Cox’s Bazar has nicely outlined. We added the reference (number 18) in the revised version of the manuscript.

The culmination of the study seems to rest on the five challenges identified; as it stands, it could be argued that these challenges could have been identified by analysing secondary data at a much more lower cost.

**Response:** Many thanks for this comment. Most of the secondary data is related to the servcie delivery, therefore analyzing secondary data might only be useful to draw some conclusion on the outcome of the programatic activities. BRAC University research team has experienced few challenges which was only possible to capture through interviewing them. Moreover, visiting the camp by the research team (three authors of this article) was helpful to get a real sense of the situation.

Where this study would find its strength is in providing specific solutions for these challenges. A prime example would be in recognising the potential of the doctors in the primary care facilities as both as a translator and as researchers in the field.

**Response:** That’s a great suggestion. However, in the camp setting most of the doctors do not speak Rohingya language and they often use translator while providing services to Rohingya community. Otherwise, it would be been a great approach. We would consider this and try to test in future IR initiative.

***** Reviewer #2:**

[See attachment]

Michael Dewey

*** Reviewer #3:

Thank you for the opportunity to review the manuscript entitled, "Embedded implementation research in humanitarian settings: Learnings from an experience with the Rohingya emergency in Cox's Bazar, Bangladesh."  The article has the potential to provide an important contribution as it focuses on the intersection of implementation research and humanitarian contexts, where there is a dearth of existing research.

My main comments focus on the framing, methods and results, and I think these points require considerable attention to maximize the potential of the paper.

Framing:

- It seems that this is a study OF embedded implementation research, and that the methods used for the study OF embedded implementation research were not necessarily "embedded."  It is important to assert more clearly how this study fits with the broader IR study, as it is not clear from the text. It is clear that the authors conducted interviews and observations, but not clear if that is separate and in addition to the broader IR study, with the clear intention of investigating the IR process, or something else. It also does not seem that the present study is itself an embedded IR, and if so the purpose and choice of methods should be clearly stated.

**Response:** In the revised version we have added a reference (Reference number 18) of the broader IR study to clarify the methods of the broader study and the present study. Present study is not an embedded IR and instead it describes the challenges of conducting embedded IR in the context of Rohingya refugee camp in Cox’s Bazar.

- Since IR--and particularly embedded IR--involves high levels of interaction, co-design, etc. with the implementors, it is important to clearly identify who the implementers are, and how they appear in the study.  (presumably the "we" is the authors, but what role do the authors play in actual service delivery?  and if people involved in direct service delivery are not authors, what role did they play?) It is reasonable to exclude the names of specific organizations or actors from the manuscript, but it is not clear who from the research team is an implementer, since the UNICEF-supported health posts are run by implementing partners, which could include BRAC, but it is unclear what role BRAC University has, or what relationship they have to the implementing partners.

**Response:** Great comment. In the revised version we have clarified about implementers and who are those in the context of Cox’s Bazar. We have added the following sentence in the ‘Introduction’ section.

“IR bridges the gap between research and policy by embedding relevant decision-makers and programme implementers (such as programme managers, front-line health workers)”.

In addition, in the methods section the following sentences highlight who are the programme implementers in the Cox’s Bazar context.

“In addition, to map the roles and responsibilities of different stakeholders we purposively selected and interviewed representatives from non-governmental organizations (NGOs), United Nations (UN) organizations and government who have been supporting and implementing MNCAH programmes in the Cox’s Bazar.”

BRAC University and BRAC (which is an NGO) did not have any relationship particularly for this research. BRAC was one of the program implementers in the camp whereas BRAC University was responsible to conduct the broader IR study (supported by UNICEF).

- Since this manuscript is distinct from the broader IR project and results, I think it needs to be clear what is the specific contribution of this study.  If it is focused on the complexity of the Rohingya crisis, that is important and valid.  However, the article seems to oscillate in its claims about what it is aiming to achieve. If it is about conducting embedded IR within the Rohingya crisis, then it seems that it should focused on the specific barriers/facilitators that enable rigorous IR.

**Response:** We have added a reference (ref number 18) in the revised version which describes the results (partly) of the broader IR study. Moreover, we have changed the title (suggested by the editor) of the manuscript to ‘ Carrying out embedded implementation research in humanitarian settings: a qualitative study in Cox's Bazar, Bangladesh ’to comply with the results of this study.

Moreover, last sentence (below) of the introduction clearly explained the aim of the present study.

“In recognition of this gap, this present study describes the challenges and key lessons learned relating to the design, implementation and management of embedded IR in humanitarian contexts based on learnings from our Cox’s Bazar experience”

Methods:

- the methods section requires strengthening. Why were qualitative methods chosen for this study?  Why Thematic Analysis? What epistemological approach was used in the design and analysis?

**Response:** We have added the following sentence in the method section with reference explaining why qualitative method was used (over quantitative) for the present study.

“For the current study, a qualitative method was applied to capture the researchers’ experience (i.e. challenges) of conducting embedded IR in the Rohingya emergency context in Cox’s Bazar”

Qualitative data can be analyzed in different ways (i.e. thematic analysis, content analysis) and thematic analysis is one of the common approaches which we applied for this study. However, we did not explain much how the analysis was done as we had word limitation of the manuscript.

Results:

- As alluded to above, all of the findings are valid and important observations of the complexity of the Rohingya crisis, but it is not clear how they inform our understanding of the feasibility of embedded IR, or how embedded IR should be implemented.  The connection from results such as "language barriers" for how that would affect IR, but the relationships between these findings and IR is not clearly or explicitly stated.

**Response:** In this research we have highlighted the challenges encountered while conducting IR in Cox’s Bazar. Language barrier is quite generic and can be applicable for other settings as well. However, in the revised version we have incorporated several comments raised by three reviewers and the editor. We hope, the revised version has more clarify on the results.

- My understanding of embedded IR is that it is, by definition, a collaborative, adaptive approach (e.g. Churruca et. al, 2019), so it is confusing to read statements such as: "It was therefore recognised that the embedded IR approach had to be flexible and able to adapt to changes identified, with research questions and methods modified accordingly." Such statements should be framed as reinforcing the need for a truly embedded approach.

**Response:** Thanks very much for this comment. Actually, our findings support the statement that embedded IR is an adaptive approach (i.e. adaption of research questions and methods depending on the situation). However, because of the unique situation (emergency) a true collaboration might not possible as most of the stakeholders are busy in providing basic health services and other emergency supplies. In that sense, truly embedded IR may not be possible in humanitarian context. We have revised the draft as below; moreover, we have added the reference (number 38) in the manuscript.

“Therefore, in the context of emergency, the embedded IR approach had to be flexible and able to adapt to changes identified, with research questions and methods modified accordingly”

**Reviewer 3:**

Shahabuddin et al January 13, 2020

This paper describes a study of the processes behind an implementation research project in Bangladesh. I was asked for a methodological report and I interpret that to include all aspects of the design and conduct of the study. The version I received did not have page numbers. My numbering has the abstract starting on page 2

1 Points of detail

Page 4 It might be helpful to define upazilas. By all means keep the word in the original too. I know we can look it up in Wikipedia of course.

**Response:** Upazilas are sub-districts. We have added that in the revised version.

Page 12 `. . . BRAC team faced several challenges while collecting data . . . ' does not seem to tell us what those challenges were.

**Response:** In the revised version of the manuscript we have restructured this sentence as below.

“Without having adequate experience working in the humanitarian context researchers may face several challenges while collecting data, identifying key stakeholders and bringing them on board to ensure the execution of the project and to ensure utilization of the research findings by key stakeholders”

Page 14 The paragraph starting `Methods of IR' does not seem to be a finding from the study but rather a generic description of IR. It could be omitted without loss of impact.

**Response:** In the revised version we have removed the mentioned sentence.

Page 15 When the authors call for an agile approach do they mean in the sense it is used in software development with roles like a scrum master or is it some other meaning? If it is the sense used in software development then it may need further explanation.

**Response:** We have revised the sentence and added a relevant reference as below;

“In addition, an adaptive and iterative approach involving relevant stakeholders may be more appropriate to understand the situation in each stage of the emergency, learning and adjusting the methodology accordingly, as required [38]”

2 Points of more substance

2.1 What is specific to IR and humanitarian setting

The authors have worked in a challenging setting and are trying to provide background help for people carrying out implementation research in humanitarian settings. But I would question how much of what they have found is limited to those settings and how much applies more generally to any type of research in any resource{poor setting. The article is quite short and more detail might help to answer my concerns. One thing I miss is any quotes from the interviewees which I feel would help to illuminate things for the reader rather than rely on the auhtors' summaries of what people said. I take the authors' five themes in turn.

**Response:** We really appreciate all comments raised by the reviewer. All are very helpful to improve the quality of the manuscript. We have tried to address all the comments in the revised version. Please find below our response for each of the comment. In addition, we have added 3 quotes in the revised version to support the results.

Page 1

Shahabuddin et al January 13, 2020

**Unique and complex context [. . . ] multiple actors**

This applies to implementation research generally I think but is it specific to humanitarian contexts?

**Response:** Every emergency situation has it’s own characteristics which are different than a normal situation. What we wanted to highlight that in humanitarian context UN organizations and other INGOs play vital role together with the government. Therefore, research teams should consider getting buy-in and ensuring involvement of those stakeholders as well together with the government.

**Adaptation to frequent changes [. . . ]**

I think anyone who has tried to carry out research based in a large organisation anywhere in the world would have faced the issue of constant changes undermining the organisation and inadvertently sabotaging the research project.

**Response:** This study highlighted the situation of an emergency context which is not stable in nature. Therefore, respondents suggested that the IR should adapt to address the ongoing/current implementation challenges and therefore, it should be iterative and adaptive in nature which we tried to explain in the discussion section.

**Difficulty accessing the camps [. . . ]**

This is true but not specific to implementation research. Any epidemiological study in rural areas with rainy seasons experiences this sort of problem. For instance in a study of a consecutive series of pregnant women and their child in rural Ethiopia our interviewers complained quite vocally about having to make visits to the more remote locations during the rainy season.

**Response:** We agree. However, findings in this study suggested to have a plan before starting the collection of data. Researchers should consider the weather, road conditions and other aspects which might affect the patient flow and their access to get in and find the appropriate respondents.

**Language and barriers to access to quality information**

This too seems to me to be a general issue. If you want to work with monoglot speakers of language X you have to learn language X or enlist staff who do speak it. To be fair I have read authors claiming as an advantage that they hired people who spoke the local language so perhaps it is not as obvious to others as it is to me.

**Response:** We agree that language problem is a general issue. However, researchers who worked in Cox’s Bazar highlighted that as a problem as well. Moreover, they emphasized on the importance of proper training of the interviewers who speak the language. Because it is not always feasible to find researchers/interviewers who are good in conducting qualitative interviews and in that case a dedicated training plan for those interviewers might help.

**Need for experienced in country research partners**

Again this is quite general. Things are slowly changing but I can remember the days when I received articles to review with not a single in-country author. Even for laboratory studies this seems a drawback. The point is still worth making as change is slow but again I question how specific it is to the context which the authors are working in.

**Response:** Great comment. We agree that often in-country researchers are ignored. However, in this paper we wanted to emphasize on that issue again. Especially, in the context of an emergency, it is critical to involve in-country researchers and they are usually familiar with the social, political and geographical context of such settings which are important to consider while conducting research.

Page 2

Shahabuddin et al January 13, 2020

2.2 What exactly was the interview content for all the

stakeholders?

I am not clear what the interviews with the NGO were intended to reveal, what their content was, and what the outcome is. Most of the findings seem to come from the work with the BRAC team. This does seem another area where some example quotes might help us to understand what happened.

**Response:** In the revised version we added the following sentence to clarify the content of the questions asked to the representatives of NGOs, and UN Organizations. Moreover, we added 3 additional quotes to support the results.

“Questions were asked to document the geographic location of the camps, living conditions of the Rohingya refugees, MNCAH service delivery modalities and related policies and roles and responsibilities of different stakeholders”

3 Summary

I have no problems with the advice the authors give and I am sure it will ring true with many people who have worked in low resource settings but I wonder how specific it is to implementation research in humanitarian settings.

**Response:** We appreciate all the comments raised by the reviewer which really helped to improve the manuscript. In our knowledge carrying out of implementation research in humanitarian context is relatively new. We agree on the fact that few findings of this study might be applicable for low resource settings. However, we believe, several issues (i.e. frequent change in service delivery modality, unique context, need of an adaptive and iterative approach etc.) highlighted in this paper are particularly applicable to humanitarian settings and would help to those researchers who plan to conduct IR in such setting.
